# Supplementary material for: Mitochondrial DNA: Hotspot for Potential Gene Modifiers Regulating Hypertrophic Cardiomyopathy
Source: J Clin Med. 2020 Jul 23;9(8):2349. doi: 10.3390/jcm9082349 (PMC7463557; doi:10.3390/jcm9082349)
Supplement: Supplementary file 1 [file jcm-09-02349-s001.pdf]

Journal of Clinical Medicine

MDPI

Supplementary Data

Mitochondrial DNA: hotspot for potential gene modifiers regulating hypertrophic cardiomyopathy

Parisa K. Kargaran[1], Jared M. Evans[2], Sara E. Bodbin[3], James G. W. Smith[4], Timothy J. Nelson[5], Chris Denning[3,*] and Diogo Mosqueira[3,*]

[1] Department of Cardiovascular Medicine, Center for Regenerative Medicine, Mayo Clinic, Rochester, MN, USA.
[2] Department of Health Science Research, Division of Biomedical Statistics and Informatics, Mayo Clinic, Rochester, MN, USA
[3] Division of Cancer & Stem Cells, Biodiscovery Institute, University of Nottingham, NG7 2RD, Nottingham, UK.
[4] Faculty of Medicine and Health Sciences, Norwich Medical School, University of East Anglia, UK.
[5] Division of General Internal Medicine, Division of Pediatric Cardiology, Departments of Medicine, Molecular Pharmacology, and Experimental Therapeutics, Mayo Clinic Center for Regenerative Medicine, Rochester, MN, USA
* Correspondence: chris.denning@nottingham.ac.uk (CD); diogo.mosqueira@nottingham.ac.uk (DM)

Supplementary material contents:

8 Tables

Supplementary Table S1- mtDNA variants identified by NGS in Family A composed by healthy and HCM individuals bearing the p.E99K-ACTC1 mutation.

Patients displaying more severe phenotypes/ clinical features are labelled in red; healthy or less severe patients are highlighted in green

Fibroblast sample	Loci	mtDNA Mutation	% Heteroplasmy	Polyphen prediction (score)
E99K1	MT-HV2, MT-OHR	m.152T>C	99.8%	N/A (non-coding)
6;7	MT-HV2, MT-OHR	m.185G>A	98.1%; 98.8%	N/A (non-coding)
E99K1; 6; 7	MT-HV2, MT-OHR, MT-CSB2	m.309_310insCT	31.7%; 32.3%; 35.8%	N/A (non-coding)
E99K1; E99K2; NC; 6; 7	MT-HV2, MT-OHR, MT-CSB2	m.310T>C	53.9%; 13.5%; 17.2%; 57.0%; 58.8%	N/A (non-coding)
E99K1; E99K2; NC; 6; 7	MT-HV2, MT-OHR, MT-CSB2	m.310_311insC	33.3%; 65.8%; 62.5%; 32.5%; 31.7%	N/A (non-coding)
6	MT-OHR, MT-LSP	m.414T>G	18.7%	N/A (non-coding)
6;7	MT-ND2	m.4659G>A	99.7%; 99.3%	Benign (0.029)
E99K1; E99K2; NC; 6; 7	MT-TA	m.5597A>C	7.5%; 7.9%; 5.1%; 12.2%; 12.4%	N/A (non-coding)
E99K1	MT-CO1	m.7109C>T	91.4%	N/A (redundant)
E99K2; NC	MT-ATP6	m.8952T>C	99.9%; 99.8%	N/A (redundant)
6; 7	MT-ATP6	m.9025G>A	99.6%; 99.6%	Probably damaging (1.000)
E99K1	MT-ATP6	m.9116T>C	99.7%	Benign (0.000)
E99K1	MT-ND4	m.11176G>A	99.6%	N/A (redundant)
6; 7	MT-ND4	m.11215C>T	99.7%; 99.6%	N/A (redundant)
E99K2; NC	MT-ND5	m.12715A>G	99.8%; 99.8%	Probably damaging (0.991)
6; 7	MT-ND5	m.12810A>G	99.9%; 99.9%	Probably damaging (STOP)
6 ;7	MT-CYB	m.15586T>C	99.8%; 99.9%	N/A (redundant)
E99K1	MT-HV1, MT-TAS	m.16168C>T	44.2%	N/A (non-coding)
6; 7	MT-HV1	m.16209T>C	99.9%; 99.9%	N/A (non-coding)

Supplementary Table S2 - mtDNA variants identified by NGS in Family B composed by healthy and HCM individuals bearing the p.E99K-ACTC1 mutation.

Patients displaying more severe phenotypes/ clinical features are labelled in red; healthy or less severe patients are highlighted in green.

Fibroblast sample	Loci	mtDNA Mutation	% heteroplasmy	Polyphen score
4;5	MT-HV2, MT-OHR	m.152T>C	99.8%; 99.8%	N/A (non-coding)
4; 5; 13; 14; 15; 16; 17; 18	MT-HV2, MT-OHR, MT-CSB2	m.310T>C	10.5%; 14.3%; 13.2%; 15.0%; 13.5%; 18.4%; 15.2%; 12.9%	N/A (non-coding)
4; 5; 13;14; 15; 16; 17; 18	MT-HV2, MT-OHR, MT-CSB2	m.310_311insC	67.6%; 67.2%; 67.3%; 65.7%; 68.6%; 57.7%; 65.0%; 67.8%	N/A (non-coding)
13	MT-HV3	m.513G>A	13.0%	N/A (non-coding)
14; 15; 16; 17; 18	MT-HV3	m.514_515delCA	56.3%; 59.4%; 55.6%; 55.0%; 55.4%	N/A (non-coding)
13	MT-HV3	m.513_514insCACA	32.5%	N/A (non-coding)
18	MT-HV3, MT-TFH	m.540A>C	11.0%	N/A (non-coding)
14; 15; 16; 17; 18	MT-HV3, MT-HSP1	m.567A>G	99.3%; 99.2%; 99.2%; 99.5%; 99.5%	N/A (non-coding)
15	MT-HV3	m.574A>G	12.3%	N/A (non-coding)
4; 5; 13; 14; 15; 16	MT-TA	m.5597A>C	25.7%; 10.5%; 14.0%; 23.8%; 13.8%; 16.8%	N/A (non-coding)
4	MT-ATP8	m.8490T>A	10.0%	Possibly damaging (0.711)
4; 5; 14	MT-CO3	m.9716T>C	100%; 99.9%; 2.4%	N/A (redundant)
4; 5; 14	MT-ND4	m.11914G>A	99.8%; 99.4%; 1.9%	N/A (redundant)
14; 15; 16; 17; 18	MT-ND4	m.11950A>G	97.9%; 99.8%; 99.8%; 99.8%; 99.9%	N/A (redundant)
4	MT-CYB	m.15639T>C	18.4%	Probably damaging (0.998)
4; 5; 14	MT-CYB	m.15670T>C	99.8%; 99.9%; 2.1%	N/A (redundant)
4	MT-HV1	m.16390G>A	12.7%	N/A (non-coding)

Supplementary Table S3 - mtDNA variants identified by NGS in Family C composed by healthy and HCM individuals bearing the p.E99K-ACTC1 mutation.

Patients displaying more severe phenotypes/ clinical features are labelled in red; healthy or less severe patients are highlighted in green.

Fibroblast sample	Loci	mtDNA Position	% heteroplasmy	Polyphen prediction (score)
8; 9; 10	MT-HV2, MT-OHR, MT-CSB2	m.310T>C	16.0%; 11.0%; 14.0%	N/A (non-coding)
8; 9; 10	MT-HV2, MT-OHR, MT-CSB2	m.310_311insC	67.1%; 69.6%; 70.1%	N/A (non-coding)
8; 9; 10	MT-HV3	m.513G>A	11.9%;14.8%; 9.9%	N/A (non-coding)
8; 9; 10	MT-HV3	m.513_514insCACA	31.9%; 33.3%; 34.9%	N/A (non-coding)
8; 10	MT-TA	m.5597A>C	11.2%; 10.7%	N/A (non-coding)

Supplementary Table S4 – Analysis of m.152T>C mtDNA variant frequency in haplogroups (number between brackets) using full length sequence set (left) or control region sequence set (right) deposited in MITOMAP database.

Using Full Length sequence set

Lineage	Top Level HG	Top Level HG Branch (ltr-num)	HG Branch (ltr-num-ltr)
L 3913 (63.2%)	L0 1159 (68.3%)	L0 1159 (68.3%)	L0a 405 (58.5%)
			L0d 582 (70.7%)
			L0f 40 (80.0%)
			L0g 9 (90.0%)
			L0k 121 (100.0%)
	L1 792 (90.2%)	L1 792 (90.2%)	L1b 266 (91.1%)
			L1c 526 (89.8%)
	L2 1212 (91.7%)	L2 1207 (91.3%)	L2a 994 (94.6%)
			L2b 92 (88.5%)
			L2c 108 (88.5%)
			L2e 12 (70.6%)
	L3	L3	L3a 27 (93.1%)
			L3d 361 (90.5%)
			L3k 13 (72.2%)
	L5 31 (79.5%)	L5 31 (79.5%)	L5a 29 (93.5%)
	L6 10 (83.3%)	L6 10 (83.3%)	
M 2251 (21.0%)	D	D1	D1j 20 (95.2%)
		D4	D4a 154 (99.4%)
			D4h 81 (69.8%)

Using Control Region sequence set

Lineage	Top Level HG	Top Level HG Branch (ltr-num)	HG Branch (ltr-num-ltr)
L 4405 (52.5%)	L0	L0	L0f 50 (71.4%)
			L0k 70 (69.3%)
	L1 1057 (88.3%)	L1 1057 (88.3%)	L1b 523 (87.5%)
			L1c 534 (89.4%)
	L2 1763 (82.0%)	L2 1755 (81.6%)	L2a 1265 (83.8%)
			L2b 225 (89.3%)
			L2c 234 (84.2%)
			L2e 28 (68.3%)
	L3	L3	L3a 21 (56.8%)
			L3d 388 (72.7%)
			L3k 16 (66.7%)
M 2598 (11.1%)	C	C4	C4 54 (100.0%)
			C4e 12 (66.7%)
	D	D4	D4a 306 (57.3%)
		D5	D5 16 (76.2%)
			D5c 24 (85.7%)
		D6	D6c 44 (100.0%)
	M	M3	M3c 43 (67.2%)
		M4	M4b 9 (50.0%)

		D5	D5c 18 (100.0%)
		D6	D6c 12 (100.0%)
	M	M2	M2b 98 (96.1%)
		M3	M3c 38 (82.6%)
		M13 48 (71.6%)	M13a 38 (97.4%)
			M13c 10 (76.9%)
		M20 54 (98.2%)	M20 54 (98.2%)
		M23 224 (96.1%)	M23 224 (96.1%)
		M24 25 (56.8%)	M24a 24 (96.0%)
		M26 10 (100.0%)	M26 10 (100.0%)
		M27	M27b 63 (100.0%)
		M28 57 (96.6%)	M28a 52 (96.3%)
		M29 13 (100.0%)	
		M36 29 (76.3%)	M36d 27 (96.4%)
		M37 14 (66.7%)	M37 10 (90.9%)
		M45 26 (92.9%)	M45a 19 (100.0%)
		M57 10 (71.4%)	
		M61 18 (69.2%)	M61 9 (52.9%)
		M68 14 (58.3%)	M68a 14 (60.9%)
		M76	M76 8 (50.0%)
	Z 191 (94.1%)	Z1 88 (100.0%)	Z1a 86 (100.0%)
		Z2 10 (100.0%)	Z2 10 (100.0%)
		Z3 61 (85.9%)	Z3 17 (70.8%)
			Z3a 21 (95.5%)

		M13	M13 59 (100.0%)
			M13c 19 (76.0%)
		M23 23 (74.2%)	M23 23 (74.2%)
		M24	M24a 13 (86.7%)
		M30	M30b 14 (100.0%)
		M36 19 (63.3%)	M36a 13 (100.0%)
		M37 18 (56.2%)	M37 17 (89.5%)
		M57 14 (93.3%)	
		M74	M74 7 (50.0%)
		M75 16 (61.5%)	M75 16 (61.5%)
	Z 311 (55.0%)	Z3 58 (93.5%)	Z3 17 (100.0%)
			Z3a 34 (89.5%)
		Z4 74 (64.9%)	Z4 27 (100.0%)
			Z4a 47 (54.0%)
N 5863 (14.1%)	A	A2	A2v 12 (80.0%)
		A5	A5c 10 (58.8%)
		A6 23 (92.0%)	A6b 16 (88.9%)
		A7 14 (63.6%)	A7 14 (63.6%)
		A11	A11b 14 (100.0%)
		A14 112 (100.0%)	A14 112 (100.0%)
		A15 152 (93.3%)	A15 141 (98.6%)
			A15b 11 (55.0%)
		A16 8 (61.5%)	A16 8 (61.5%)
		A24 10 (100.0%)	A24 10 (100.0%)

			Z3b 8 (80.0%)
		Z4 26 (100.0%)	Z4a 17 (100.0%)
N 7119 (21.4%)	A	A	A 57 (86.4%)
		A1 13 (100.0%)	A1a 12 (100.0%)
		A2	A2v 10 (90.9%)
		A6 16 (100.0%)	
		A8	A8a 10 (62.5%)
		A11 34 (97.1%)	A11a 22 (95.7%)
			A11b 10 (100.0%)
		A12 12 (100.0%)	A12a 10 (100.0%)
		A13 19 (100.0%)	A13 19 (100.0%)
		A14 34 (94.4%)	A14 34 (94.4%)
		A15 67 (97.1%)	A15a 10 (100.0%)
			A15c 53 (98.1%)
		A17 40 (95.2%)	A17 40 (95.2%)
	B	B2	B2q 15 (71.4%)
	F	F1	F1b 93 (66.9%)
			F1c 46 (97.9%)
		F3 162 (54.9%)	F3b 155 (68.6%)
		F4	F4a 18 (56.2%)
	H	H1	H1i 17 (89.5%)
		H2	H2b 28 (96.6%)
		H3	H3g 58 (96.7%)
			H3k 17 (100.0%)
			H3q 6 (54.5%)
		H8	H8c 22 (95.7%)

		A25 7 (70.0%)	A25 7 (70.0%)
	B	B2	B2b 83 (85.6%)
			B2u 6 (60.0%)
	F	F2	F2i 14 (58.3%)
	H	H9 22 (100.0%)	H9a 22 (100.0%)
		H32 62 (100.0%)	H32 62 (100.0%)
		H36 12 (70.6%)	H36 12 (70.6%)
	HV	HV1	HV1b 23 (50.0%)
		HV2 58 (65.9%)	HV2a 38 (84.4%)
	I	I2 27 (71.1%)	I2 24 (80.0%)
		I3 11 (68.8%)	I3a 11 (68.8%)
	J	J1	J1d 84 (52.2%)
		J2 219 (58.1%)	J2b 126 (84.0%)
	K	K1	K1c 145 (91.2%)
		K2	K2a 50 (69.4%)
	N	N1	N1b 123 (52.1%)
	R	R2 63 (61.8%)	R2 52 (61.9%)
			R2b 10 (76.9%)
		R7 15 (51.7%)	R7 9 (81.8%)
	S	S	S 22 (100.0%)
	T	T2	T2d 20 (69.0%)
	U	U2	U2c 45 (68.2%)
			U2e 170 (63.7%)
		U7 269 (59.1%)	U7a 208 (79.1%)
		U8	U8c 8 (61.5%)
	X	X4 8 (72.7%)	X4 8 (72.7%)

		H9 16 (88.9%)	H9a 14 (87.5%)
		H16	H16a 10 (100.0%)
			H16d 10 (100.0%)
		H23 58 (65.9%)	H23 58 (65.9%)
		H34 10 (90.9%)	H34 10 (90.9%)
		H36 20 (76.9%)	H36 20 (76.9%)
		H46 15 (78.9%)	H46 8 (72.7%)
		H52 12 (92.3%)	H52 12 (92.3%)
	HV	HV1	HV1b 32 (64.0%)
		HV2 40 (88.9%)	HV2a 34 (91.9%)
		HV9	HV9a 12 (100.0%)
		HV12	HV12a 7 (58.3%)
	I	I2 153 (91.1%)	I2 93 (93.0%)
			I2a 22 (84.6%)
			I2c 13 (86.7%)
			I2d 20 (95.2%)
		I3 64 (98.5%)	I3a 35 (97.2%)
			I3d 12 (100.0%)
	J	J1	J1d 93 (94.9%)
		J2 575 (86.6%)	J2a 253 (77.1%)
			J2b 322 (95.8%)
	K	K1	K1c 216 (95.6%)
		K2 188 (62.5%)	K2a 184 (85.6%)
	N	N1 201 (67.2%)	N1b 129 (92.1%)
		N7 8 (57.1%)	
		N8 13 (81.2%)	N8 13 (81.2%)
		N11 14 (58.3%)	N11b 14 (93.3%)
	P	P1	P1 15 (78.9%)
		P5 24 (85.7%)	P5 24 (85.7%)
		P9 7 (63.6%)	

	R	R2 65 (95.6%)	R2 27 (100.0%)
			R2b 12 (92.3%)
			R2d 15 (88.2%)
		R5 38 (76.0%)	R5a 38 (77.6%)
		R21 12 (100.0%)	R21 12 (100.0%)
	S 26 (53.1%)	S1 13 (59.1%)	S1 13 (92.9%)
	T	T1 413 (67.4%)	T1a 409 (72.8%)
		T2	T2c 113 (59.2%)
			T2n 10 (100.0%)
	U	U2 358 (81.7%)	U2b 24 (52.2%)
			U2c 19 (95.0%)
			U2d 24 (100.0%)
			U2e 286 (89.9%)
		U7 442 (98.7%)	U7 25 (96.2%)
			U7a 307 (99.7%)
			U7b 110 (96.5%)
	X	X4 9 (90.0%)	X4 9 (90.0%)

Supplementary Table S5 - Analysis of m.309_310insCT mtDNA variant frequency in haplogroups (number between brackets) using full length sequence set (left) or control region sequence set (right) deposited in MITOMAP database.

Using Full Length sequence set			
**Lineage**	**Top Level HG**	**Top Level HG Branch (ltr-num)**	**HG Branch (ltr-num-ltr)**
L 1001 (16.2%)	L6 7 (58.3%)	L6 7 (58.3%)	
M 3220 (30.1%)	C	C	C 243 (54.0%)
		C1 161 (51.6%)	C1b 65 (51.6%)
			C1c 32 (54.2%)
			C1d 61 (50.8%)
	D	D1	D1j 12 (57.1%)
		D2 50 (58.1%)	D2a 41 (65.1%)
			D2b 9 (60.0%)
		D4	D4s 9 (69.2%)
	M	M1	M1b 15 (50.0%)
		M5	M5b 16 (57.1%)
		M11 19 (50.0%)	M11b 7 (70.0%)
		M13	M13c 8 (61.5%)
		M21	M21a 18 (54.5%)
		M33	M33a 27 (67.5%)
		M34 6 (60.0%)	
		M35	M35b 41 (71.9%)
		M36 30 (78.9%)	M36d 23 (82.1%)

Using Control Region sequence set			
**Lineage**	**Top Level HG**	**Top Level HG Branch (ltr-num)**	**HG Branch (ltr-num-ltr)**
L 1640 (19.5%)	L4	L4	L4 6 (50.0%)
M 4047 (17.3%)	C	C4	C4e 9 (50.0%)
	D	D2 129 (64.2%)	D2c 127 (72.2%)
		D4	D4 22 (59.5%)
		D5	D5c 16 (57.1%)
		D6	D6c 25 (56.8%)
	M	M5	M5c 14 (51.9%)
		M10	M10 8 (72.7%)
		M11	M11 17 (70.8%)
		M13	M13 46 (78.0%)
			M13c 20 (80.0%)
		M38 6 (50.0%)	
		M65	M65b 7 (58.3%)
		M74	M74 8 (57.1%)
		M91	M91a 7 (50.0%)
	Q	Q1	Q1a 37 (50.0%)
	Z	Z3 34 (54.8%)	Z3a 23 (60.5%)
		Z4	Z4 17 (63.0%)

		M38 13 (56.5%)	
		M52	M52b 8 (57.1%)
		M57 10 (71.4%)	
		M62	M62b 10 (55.6%)
		M73	M73 6 (54.5%)
N 9019 (27.1%)	A	A2	A2a 118 (52.2%)
			A2b 22 (51.2%)
			A2p 7 (50.0%)
			A2w 11 (50.0%)
			A2y 12 (80.0%)
		A5 63 (60.0%)	A5a 53 (80.3%)
		A13 11 (57.9%)	A13 11 (57.9%)
	B	B2	B2d 7 (58.3%)
			B2l 7 (50.0%)
	H	H1	H1h 11 (55.0%)
			H1r 6 (60.0%)
			H1v 8 (50.0%)
		H7	H7a 29 (52.7%)
			H7e 16 (66.7%)
		H10	H10e 39 (61.9%)
		H31	H31a 10 (76.9%)
		H35 14 (60.9%)	H35 11 (55.0%)
		H40 7 (53.8%)	
		H49	H49a 12 (57.1%)
		H51 6 (54.5%)	
		H52 8 (61.5%)	H52 8 (61.5%)

N 6358 (15.3%)	A	A2	A2g 10 (71.4%)
			A2m 12 (66.7%)
		A11	A11b 8 (57.1%)
		A24 7 (70.0%)	A24 7 (70.0%)
	HV	HV14 22 (62.9%)	HV14 22 (62.9%)
	S	S	S 11 (50.0%)
	W	W3 34 (61.8%)	W3a 32 (60.4%)

	HV	HV0	HV0d 7 (53.8%)
		HV4 56 (58.3%)	HV4a 47 (67.1%)
		HV9	HV9 8 (53.3%)
		HV14 46 (80.7%)	HV14a 45 (81.8%)
		HV16 10 (66.7%)	HV16 10 (66.7%)
	I	I1	I1 11 (52.4%)
			I1b 19 (51.4%)
			I1c 7 (50.0%)
		I2	I2a 13 (50.0%)
		I3 38 (58.5%)	I3a 23 (63.9%)
			I3d 6 (50.0%)
	N	N2 7 (63.6%)	N2a 7 (63.6%)
		N7 8 (57.1%)	
	P	P9 7 (63.6%)	
	R	R2	R2d 10 (58.8%)
		R8 64 (63.4%)	R8b 42 (87.5%)
	T	T1	T1b 25 (62.5%)
		T2	T2n 7 (70.0%)
	U	U7	U7b 61 (53.5%)
		U8	U8a 57 (61.3%)
		U9 9 (56.2%)	
	V	V5 7 (70.0%)	V5 7 (70.0%)
		V10 15 (62.5%)	V10a 9 (81.8%)
		V12 6 (60.0%)	V12 6 (60.0%)
		V22 6 (50.0%)	V22 6 (50.0%)
	W	W1	W1b 15 (57.7%)
		W3 72 (52.2%)	W3a 46 (50.0%)
			W3b 25 (58.1%)
		W6 46 (54.1%)	W6a 11 (55.0%)
	X	X2	X2p 6 (60.0%)

Supplementary Table S6 - Analysis of m.309_310insCCT mtDNA variant frequency in haplogroups (number between brackets) using full length sequence set (left) or control region sequence set (right) deposited in MITOMAP database.

Using Full Length sequence set			
**Lineage**	**Top Level HG**	**Top Level HG Branch (ltr-num)**	**HG Branch (ltr-num-ltr)**
M 689 (6.4%)	M	M50 9 (50.0%)	M50a 8 (61.5%)
		M53 17 (65.4%)	M53b 16 (69.6%)

Using Control Region sequence set			
**Lineage**	**Top Level HG**	**Top Level HG Branch (ltr-num)**	**HG Branch (ltr-num-ltr)**
N 2645 (6.4%)	B	B2	B2y 30 (54.5%)
	H	H66 13 (50.0%)	H66a 13 (50.0%)

Supplementary Table S7 - Analysis of m.310T>C mtDNA variant frequency in haplogroups (number between brackets) using full length sequence set (left) or control region sequence set (right) deposited in MITOMAP database.

Using Full Length sequence set			
**Lineage**	**Top Level HG**	**Top Level HG Branch (ltr-num)**	**HG Branch (ltr-num-ltr)**
L 2092 (33.8%)	L2	L2	L2e 9 (52.9%)
	L6 7 (58.3%)	L6 7 (58.3%)	
M 4436 (41.4%)	C	C	C 297 (66.0%)
		C1 187 (59.9%)	C1b 74 (58.7%)
			C1c 44 (74.6%)
			C1d 66 (55.0%)
		C4	C4 15 (51.7%)
			C4c 23 (69.7%)
	D	D1	D1j 13 (61.9%)
		D2 53 (61.6%)	D2a 42 (66.7%)
			D2b 10 (66.7%)
		D4	D4 36 (51.4%)
			D4a 88 (56.8%)
			D4b 136 (55.3%)
			D4g 44 (54.3%)
			D4o 29 (54.7%)
			D4s 9 (69.2%)
		D6	D6c 7 (58.3%)

Using Control Region sequence set			
**Lineage**	**Top Level HG**	**Top Level HG Branch (ltr-num)**	**HG Branch (ltr-num-ltr)**
L 1878 (22.4%)	L4	L4	L4 7 (58.3%)
M 6220 (26.6%)	C	C4	C4e 10 (55.6%)
		C7	C7 21 (50.0%)
	D	D1	D1 63 (56.2%)
			D1a 17 (85.0%)
			D1e 19 (51.4%)
			D1f 19 (59.4%)
		D2 178 (88.6%)	D2c 176 (100.0%)
		D4	D4 26 (70.3%)
			D4g 91 (54.2%)
		D5	D5 11 (52.4%)
			D5c 21 (75.0%)
		D6	D6c 30 (68.2%)
	G	G	G 24 (77.4%)
	M	M3	M3c 33 (51.6%)
		M5	M5c 15 (55.6%)
		M9	M9 159 (68.2%)
		M10	M10 9 (81.8%)

	M	M1 116 (53.7%)	M1a 87 (51.5%)
			M1b 23 (76.7%)
		M3	M3c 31 (67.4%)
		M5 92 (50.3%)	M5a 66 (50.0%)
			M5b 19 (67.9%)
		M6 13 (52.0%)	M6a 11 (52.4%)
		M7	M7a 59 (58.4%)
		M8 54 (60.0%)	M8a 53 (59.6%)
		M11 24 (63.2%)	M11a 13 (56.5%)
			M11b 7 (70.0%)
		M12	M12b 30 (69.8%)
		M13	M13c 9 (69.2%)
		M17	M17c 20 (50.0%)
		M21 45 (55.6%)	M21a 19 (57.6%)
			M21b 26 (54.2%)
		M24	M24a 13 (52.0%)
		M33 46 (52.9%)	M33a 28 (70.0%)
			M33c 12 (57.1%)
		M34 7 (70.0%)	
		M35 51 (54.3%)	M35b 44 (77.2%)
		M36 33 (86.8%)	M36d 26 (92.9%)
		M37 12 (57.1%)	
		M38 15 (65.2%)	
		M39 25 (78.1%)	M39b 18 (94.7%)

		M11	M11 19 (79.2%)
		M13	M13 47 (79.7%)
			M13c 20 (80.0%)
		M18	M18a 14 (51.9%)
		M35	M35b 25 (56.8%)
		M38 6 (50.0%)	
		M42	M42b 8 (50.0%)
		M45	M45 5 (50.0%)
		M62	M62 10 (55.6%)
			M62a 38 (70.4%)
		M65	M65b 8 (66.7%)
		M66	M66b 8 (50.0%)
		M73	M73 15 (53.6%)
		M74	M74 11 (78.6%)
		M91	M91a 8 (57.1%)
	Q	Q1	Q1a 42 (56.8%)
			Q1c 12 (100.0%)
	Z	Z3 45 (72.6%)	Z3 10 (58.8%)
			Z3a 30 (78.9%)
		Z4	Z4 19 (70.4%)
N 12242 (29.5%)	A	A2	A2g 12 (85.7%)
			A2m 12 (66.7%)
			A2v 11 (73.3%)
		A6	A6b 10 (55.6%)

		M42	M42b 10 (55.6%)
		M44 8 (66.7%)	M44a 7 (63.6%)
		M45 18 (64.3%)	M45a 11 (57.9%)
		M46 10 (83.3%)	
		M50 15 (83.3%)	M50a 12 (92.3%)
		M52	M52b 11 (78.6%)
		M53 23 (88.5%)	M53b 22 (95.7%)
		M54 6 (54.5%)	M54 6 (54.5%)
		M57 11 (78.6%)	
		M61 14 (53.8%)	
		M62 18 (81.8%)	M62b 15 (83.3%)
		M71 48 (51.6%)	M71a 27 (62.8%)
		M72 10 (52.6%)	M72a 9 (60.0%)
		M73 14 (56.0%)	M73 8 (72.7%)
		M76 15 (60.0%)	M76 13 (81.2%)
		M91 16 (69.6%)	M91a 12 (66.7%)
	Z	Z1 50 (56.8%)	Z1a 49 (57.0%)
		Z3	Z3a 17 (77.3%)
		Z4	Z4a 9 (52.9%)
N 13904 (41.8%)	A 776 (55.6%)	A2 546 (61.0%)	A2 198 (55.9%)
			A2a 163 (72.1%)
			A2b 26 (60.5%)
			A2d 15 (60.0%)
			A2g 11 (84.6%)
		A11	A11b 9 (64.3%)
		A15 109 (66.9%)	A15 99 (69.2%)
			A15b 10 (50.0%)
		A24 8 (80.0%)	A24 8 (80.0%)
	B	B2 384 (61.8%)	B2 11 (68.8%)
			B2a 119 (74.8%)
			B2b 63 (64.9%)
			B2c 64 (69.6%)
			B2y 51 (92.7%)
		B4	B4e 10 (62.5%)
			B4m 42 (66.7%)
		B6	B6 8 (53.3%)
	F	F	F 17 (68.0%)
		F2	F2i 12 (50.0%)
	H	H1	H1u 10 (71.4%)
		H5	H5b 25 (65.8%)
			H5n 5 (50.0%)
		H7	H7i 8 (57.1%)
		H14	H14b 34 (50.0%)
		H41 15 (78.9%)	H41a 15 (78.9%)
		H55 26 (76.5%)	H55b 26 (76.5%)
		H57 88 (59.1%)	H57 88 (59.1%)
		H66 20 (76.9%)	H66a 20 (76.9%)
		H101 29 (82.9%)	H101 29 (82.9%)

			A2h 11 (64.7%)
			A2j 7 (70.0%)
			A2p 12 (85.7%)
			A2q 10 (52.6%)
			A2v 9 (81.8%)
			A2w 15 (68.2%)
			A2y 13 (86.7%)
		A5 69 (65.7%)	A5a 56 (84.8%)
		A11	A11b 5 (50.0%)
		A13 13 (68.4%)	A13 13 (68.4%)
		A15	A15a 5 (50.0%)
	B	B2 589 (68.5%)	B2 231 (64.9%)
			B2a 36 (76.6%)
			B2b 138 (72.3%)
			B2c 34 (73.9%)
			B2d 12 (100.0%)
			B2e 21 (58.3%)
			B2i 23 (69.7%)
			B2l 12 (85.7%)
			B2o 14 (51.9%)
			B2q 19 (90.5%)
			B2y 10 (71.4%)
		B4	B4d 19 (59.4%)
			B4e 8 (57.1%)

	HV	HV0	HV0f 11 (61.1%)
		HV14 33 (94.3%)	HV14 33 (94.3%)
	I	I1	I1d 12 (63.2%)
		I2 19 (50.0%)	I2 17 (56.7%)
	J	JT	JT 11 (64.7%)
	N	N10	N10b 12 (54.5%)
	P	P2 6 (60.0%)	P2 6 (60.0%)
	R	R1	R1a 8 (66.7%)
		R9	R9 252 (64.0%)
		R11	R11 34 (79.1%)
	S	S	S 13 (59.1%)
	T	T2	T2d 21 (72.4%)
	U	U1	U1 7 (63.6%)
		U2	U2 22 (53.7%)
		U4 436 (58.4%)	U4a 426 (73.2%)
	V	V	V 64 (56.6%)
	W	W3 36 (65.5%)	W3a 34 (64.2%)
	X	X2	X2e 31 (53.4%)
		X4 7 (63.6%)	X4 7 (63.6%)
	Y	Y1	Y1 63 (58.3%)

		B5	B5b 78 (50.6%)
	F	F1	F1b 77 (55.4%)
			F1e 17 (56.7%)
		F3	F3a 41 (59.4%)
	H	H1	H1h 14 (70.0%)
			H1j 75 (67.0%)
			H1n 53 (80.3%)
			H1r 9 (90.0%)
			H1v 10 (62.5%)
			H1z 8 (53.3%)
		H2 457 (55.7%)	H2a 432 (55.5%)
			H2b 19 (65.5%)
		H3	H3b 46 (63.0%)
		H5	H5b 55 (71.4%)
		H6 240 (63.7%)	H6a 214 (66.0%)
			H6b 19 (70.4%)
		H7 162 (50.8%)	H7a 42 (76.4%)
			H7c 31 (64.6%)
			H7e 23 (95.8%)
		H8 29 (53.7%)	H8c 14 (60.9%)
		H10 116 (75.8%)	H10a 21 (72.4%)
			H10e 53 (84.1%)
		H11	H11b 8 (61.5%)
		H13 203 (64.4%)	H13a 182 (65.7%)

			H13b 11 (50.0%)
			H13c 6 (60.0%)
		H14 48 (56.5%)	H14a 32 (60.4%)
			H14b 15 (55.6%)
		H15 67 (69.8%)	H15a 46 (73.0%)
			H15b 18 (64.3%)
		H17	H17a 14 (60.9%)
		H20	H20a 12 (50.0%)
		H26 37 (50.0%)	H26a 29 (58.0%)
		H30 5 (50.0%)	
		H31	H31a 12 (92.3%)
		H35 18 (78.3%)	H35 15 (75.0%)
		H39 20 (64.5%)	H39 8 (72.7%)
			H39c 7 (63.6%)
		H40 9 (69.2%)	
		H41 25 (80.6%)	H41a 25 (80.6%)
		H44 15 (68.2%)	H44b 12 (85.7%)
		H49 33 (71.7%)	H49 12 (57.1%)
			H49a 17 (81.0%)
		H51 6 (54.5%)	
		H52 10 (76.9%)	H52 10 (76.9%)
		H55 10 (50.0%)	
		H56 14 (51.9%)	H56 11 (55.0%)
		H73 6 (60.0%)	

		H101 7 (50.0%)	H101 7 (50.0%)
	HV 442 (57.0%)	HV0	HV0d 9 (69.2%)
		HV1 81 (60.9%)	HV1a 48 (64.0%)
			HV1b 30 (60.0%)
		HV2 25 (55.6%)	HV2a 21 (56.8%)
		HV4 76 (79.2%)	HV4 9 (52.9%)
			HV4a 60 (85.7%)
		HV6 12 (66.7%)	HV6 9 (60.0%)
		HV9 29 (78.4%)	HV9 14 (93.3%)
			HV9a 8 (66.7%)
		HV12	HV12a 7 (58.3%)
		HV13 9 (64.3%)	
		HV14 50 (87.7%)	HV14a 49 (89.1%)
		HV16 13 (86.7%)	HV16 13 (86.7%)
		HV18 10 (76.9%)	HV18 10 (76.9%)
	I	I	I 14 (51.9%)
		I1	I1 14 (66.7%)
			I1b 20 (54.1%)
			I1c 8 (57.1%)
			I1f 15 (71.4%)
		I2 93 (55.4%)	I2 56 (56.0%)
			I2a 18 (69.2%)
			I2d 16 (76.2%)
		I3 49 (75.4%)	I3a 28 (77.8%)
			I3d 10 (83.3%)

		I4	I4b 6 (54.5%)
	K	K1	K1b 100 (56.2%)
			K1d 8 (66.7%)
	N	N2 7 (63.6%)	N2a 7 (63.6%)
		N7 9 (64.3%)	
		N9 205 (63.1%)	N9a 180 (63.8%)
			N9b 25 (58.1%)
		N10	N10a 7 (50.0%)
		N21	N21a 6 (54.5%)
	P	P9 9 (81.8%)	
	R	R0 170 (56.5%)	R0a 151 (58.8%)
		R1 43 (60.6%)	R1a 36 (73.5%)
		R2 38 (55.9%)	R2 14 (51.9%)
			R2b 12 (92.3%)
			R2d 10 (58.8%)
		R8 70 (69.3%)	R8a 28 (52.8%)
			R8b 42 (87.5%)
		R9	R9b 68 (50.4%)
		R11	R11b 9 (75.0%)
		R21 6 (50.0%)	R21 6 (50.0%)
		R30	R30a 26 (53.1%)
	T	T1 342 (55.8%)	T1a 310 (55.2%)
			T1b 29 (72.5%)
		T2	T2 44 (51.8%)

			T2d 13 (59.1%)
			T2e 68 (58.1%)
			T2n 8 (80.0%)
	U	U2 232 (53.0%)	U2e 189 (59.4%)
		U4	U4a 166 (52.5%)
			U4b 83 (53.9%)
		U7 238 (53.1%)	U7a 161 (52.3%)
			U7b 65 (57.0%)
		U8 80 (56.7%)	U8a 67 (72.0%)
		U9 9 (56.2%)	
	V 453 (63.3%)	V1 83 (71.6%)	V1a 79 (70.5%)
		V2 26 (59.1%)	V2b 12 (85.7%)
		V3 29 (76.3%)	V3 8 (72.7%)
			V3c 11 (84.6%)
		V5 9 (90.0%)	V5 9 (90.0%)
		V7 65 (68.4%)	V7 7 (63.6%)
			V7a 55 (68.8%)
		V8 12 (75.0%)	V8 12 (75.0%)
		V9 16 (80.0%)	V9a 16 (84.2%)
		V10 20 (83.3%)	V10a 11 (100.0%)
			V10b 9 (69.2%)
		V12 8 (80.0%)	V12 8 (80.0%)
		V13 9 (90.0%)	V13 9 (90.0%)
		V15 9 (60.0%)	V15a 8 (57.1%)
		V18 11 (68.8%)	V18a 9 (69.2%)

		V22 10 (83.3%)	V22 10 (83.3%)
	W	W1	W1a 23 (53.5%)
			W1b 15 (57.7%)
		W3 83 (60.1%)	W3a 54 (58.7%)
			W3b 27 (62.8%)
		W5	W5a 21 (56.8%)
		W6 53 (62.4%)	W6 24 (51.1%)
			W6a 14 (70.0%)
	X	X2	X2a 20 (55.6%)
			X2e 29 (67.4%)
			X2i 12 (70.6%)
			X2p 7 (70.0%)
		X3 6 (50.0%)	
		X4 8 (80.0%)	X4 8 (80.0%)
	Y 70 (50.4%)	Y2 29 (53.7%)	Y2a 24 (54.5%)

Supplementary Table S8 - Analysis of m.16319G>A mtDNA variant frequency in haplogroups (number between brackets) using full length sequence set (left) or control region sequence set (right) deposited in MITOMAP database.

Using Full Length sequence set			
**Lineage**	**Top Level HG**	**Top Level HG Branch (ltr-num)**	**HG Branch (ltr-num-ltr)**
M 939 (8.8%)	D	D3 39 (100.0%)	D3 39 (100.0%)
	M	M2 232 (95.1%)	M2a 129 (99.2%)
			M2b 96 (94.1%)
		M8 88 (97.8%)	M8a 87 (97.8%)
		M32 162 (97.0%)	M32a 10 (100.0%)
			M32c 152 (99.3%)
		M35	M35a 14 (50.0%)
		M40 19 (65.5%)	M40a 19 (67.9%)
		M46 7 (58.3%)	
		M55 13 (100.0%)	M55 13 (100.0%)
		M60 9 (64.3%)	M60a 9 (90.0%)
N 1810 (5.4%)	A 1356 (97.2%)	A	A 65 (98.5%)
		A1 13 (100.0%)	A1a 12 (100.0%)
		A2 859 (96.0%)	A2 344 (97.2%)
			A2a 225 (99.6%)
			A2b 43 (100.0%)
			A2d 21 (84.0%)
			A2f 18 (94.7%)

Using Control Region sequence set			
**Lineage**	**Top Level HG**	**Top Level HG Branch (ltr-num)**	**HG Branch (ltr-num-ltr)**
L 159 (1.9%)	L4	L4	L4 7 (58.3%)
M 1344 (5.7%)	M	M2 124 (53.9%)	M2a 110 (90.9%)
			M2b 14 (66.7%)
		M8 435 (75.5%)	M8a 435 (99.1%)
		M12	M12b 82 (59.0%)
		M32 50 (98.0%)	M32c 48 (100.0%)
		M40	M40a 8 (50.0%)
		M55 22 (100.0%)	M55 22 (100.0%)
		M60 13 (54.2%)	M60a 13 (81.2%)
N 4381 (10.6%)	A 3718 (98.6%)	A	A 916 (97.7%)
		A1 29 (100.0%)	A1a 29 (100.0%)
		A2 1570 (98.6%)	A2 797 (98.8%)
			A2a 332 (98.2%)
			A2b 222 (100.0%)
			A2d 98 (100.0%)
			A2g 14 (100.0%)
			A2m 18 (100.0%)
			A2q 19 (100.0%)

			A2g 11 (84.6%)
			A2h 15 (88.2%)
			A2i 15 (88.2%)
			A2j 9 (90.0%)
			A2l 13 (100.0%)
			A2m 12 (100.0%)
			A2p 13 (92.9%)
			A2q 18 (94.7%)
			A2u 11 (100.0%)
			A2v 11 (100.0%)
			A2w 22 (100.0%)
			A2y 13 (86.7%)
		A5 104 (99.0%)	A5a 66 (100.0%)
			A5b 25 (100.0%)
		A6 16 (100.0%)	
		A8 23 (100.0%)	A8a 16 (100.0%)
		A11 35 (100.0%)	A11a 23 (100.0%)
			A11b 10 (100.0%)
		A12 12 (100.0%)	A12a 10 (100.0%)
		A13 19 (100.0%)	A13 19 (100.0%)
		A14 36 (100.0%)	A14 36 (100.0%)
		A15 69 (100.0%)	A15a 10 (100.0%)
			A15c 54 (100.0%)
		A17 41 (97.6%)	A17 41 (97.6%)

			A2u 10 (83.3%)
			A2v 14 (93.3%)
		A5 162 (99.4%)	A5a 63 (100.0%)
			A5b 82 (98.8%)
			A5c 17 (100.0%)
		A6 24 (96.0%)	A6b 18 (100.0%)
		A7 22 (100.0%)	A7 22 (100.0%)
		A8 47 (100.0%)	A8a 38 (100.0%)
		A11 575 (99.8%)	A11 561 (99.8%)
			A11b 14 (100.0%)
		A14 111 (99.1%)	A14 111 (99.1%)
		A15 161 (98.8%)	A15 141 (98.6%)
			A15b 20 (100.0%)
		A16 11 (84.6%)	A16 11 (84.6%)
		A19 54 (100.0%)	A19 54 (100.0%)
		A24 10 (100.0%)	A24 10 (100.0%)
		A25 10 (100.0%)	A25 10 (100.0%)
	N	N2 30 (53.6%)	N2a 30 (93.8%)
	P	P4 21 (63.6%)	P4a 21 (100.0%)
	R	R7 29 (100.0%)	R7 11 (100.0%)

	B	B2	B2y 8 (57.1%)
	H	H31	H31a 12 (92.3%)
	I	I1	I1c 12 (85.7%)
	N	N2 11 (100.0%)	N2a 11 (100.0%)
	R	R7 28 (100.0%)	R7a 16 (100.0%)
